# AGG/CCT interruptions affect nucleosome formation and positioning of healthy-length CGG/CCG triplet repeats

**DOI:** 10.1186/1471-2091-14-33

**Published:** 2013-11-22

**Authors:** Catherine B Volle, Sarah Delaney

**Affiliations:** 1 Department of Molecular Biology, Cell Biology and Biochemistry, Brown University, Providence, RI 02912, USA; 2 Department of Chemistry, Brown University, Providence, RI 02912, USA

**Keywords:** Trinucleotide repeats, Nucleosome, DNA positioning, Fragile X Syndrome, DNA periodicity

## Abstract

**Background:**

Fragile X Syndrome (FXS), the most common inherited form of mental retardation, is caused by expansion of a CGG/CCG repeat tract in the 5′-untranslated region of the *fragile X mental retardation* (*FMR1*) gene, which changes the functional organization of the gene from euchromatin to heterochromatin. Interestingly, healthy-length repeat tracts possess AGG/CCT interruptions every 9–10 repeats, and clinical data shows that loss of these interruptions is linked to expansion of the repeat tract to disease-length. Thus, it is important to understand how these interruptions alter the behavior of the repeat tract in the packaged gene.

**Results:**

To investigate how uninterrupted and interrupted CGG/CCG repeat tracts interact with the histone core, we designed experiments using the nucleosome core particle, the most basic unit of chromatin packaging. Using DNA containing 19 CGG/CCG repeats, flanked by either a nucleosome positioning sequence or the *FMR1* gene sequence, we determined that the addition of a single AGG/CCT interruption modulates both the ability of the CGG/CCG repeat DNA to incorporate into a nucleosome and the rotational and translational position of the repeat DNA around the histone core when flanked by the nucleosome positioning sequence. The presence of these interruptions also alters the periodicity of the DNA in the nucleosome; interrupted repeat tracts have a greater periodicity than uninterrupted repeats.

**Conclusions:**

This work defines the ability of AGG/CCT interruptions to modulate the behavior of the repeat tract in the packaged gene and contributes to our understanding of the role that AGG/CCT interruptions play in suppressing expansion and maintaining the correct functional organization of the *FMR1* gene, highlighting a protective role played by the interruptions in genomic packaging.

## Background

Fragile X Syndrome (FXS), caused by an expansion of CGG/CCG triplet repeats in the 5′-untranslated region of the *fragile X mental retardation 1* (*FMR1*) gene, is the most common form of inherited mental retardation [1-6]. Expansion of the repeat tract can occur during DNA replication and it is generally accepted that formation of non-canonical structures by the triplet repeat sequence contributes to the expansion mechanism [7-13]. It is also known that expansion of the CGG/CCG repeats leads to hyper-methylation of the repeat tract and the *FMR1* promoter, which, along with the loss of histone acetylation, causes the functional organization of *FMR1* to switch from transcriptionally active euchromatin to the tightly compacted heterochromatin, resulting in a loss of the *FMR1* protein product [3,14-16]. The loss of this protein results in transcriptional disregulation at the synapse, leading to compromised synaptic plasticity [10,17,18].

The number of CGG/CCG repeats within the *FMR1* gene is variable. The length of the repeat tract is typically defined as follows: healthy individuals have 6–54 repeats, individuals carrying a pre-mutation have 55–200 repeats, and FXS patients have over 200 repeats [2,3,6,14-16,19]. Interestingly, sequencing analysis of healthy *FMR1* alleles has shown that AGG/CCT interruptions are present every 9–10 repeats with (CGG)_9-10_AGG(CGG)_9_AGG(CGG)_9_ being the most common allele [19-24]. The importance of these interruptions is underscored by the observation that uninterrupted CGG/CCG tracts containing 34–59 repeats can expand to diseased length, yet similar repeat tracts with AGG/CCT interruptions are stably transmitted to offspring and do not expand to disease length [23,25-27]. Thus, AGG/CCT interruptions appear to play an important role in suppressing expansion of CGG/CCG triplet repeats.

Previous work has shown that AGG/CCT interruptions alter the stability of non-canonical structures formed by oligonucleotides containing CGG/CCG repeats [28,29]. Interestingly, the presence of AGG/CCT interruptions does not affect transcription of the *FMR1* gene or translation of the *FMR1* mRNA, indicating that the ability to destabilize non-canonical structures protects against DNA polymerase-mediated expansion, most likely during early embryogenesis when the pre-mutation repeat tract can expand to disease-length, and manifestation of the disease state occurs [17,30,31]. However, given the importance of AGG/CCT interruptions in maintaining the number of repeats within the CGG/CCG repeat tract, it is possible that AGG/CCT interruptions also have other protective roles besides destabilizing non-canonical structures.

Of particular interest is the possibility that these interruptions may modulate the functional organization of the *FMR1* gene, especially the interaction of the repeat DNA with the histone core. The transition from euchromatin to heterochromatin is an important step in the pathogenesis of FXS [17,32,33]; to understand that transition we must first understand how the repeat tract behaves in the packaged genome and how that behavior is influenced by the presence of interruptions. Previous work has focused primarily on either pre-mutation or disease-length repeat tracts and/or uninterrupted repeats. Furthermore, most research that investigates healthy-length repeats or interrupted repeats has focused solely on the ability of the DNA to form chromatin while little attention has been paid to the DNA structure in the chromatin formed [33-37]. Here, we have designed experiments using nucleosome core particles, the most basic unit of chromatin packaging, composed of 146 base pairs (bps) of DNA wrapped around a histone octamer [38]. Using DNA containing 19 CGG/CCG repeats, flanked by either the nucleosome positioning sequence S1 or the *FMR1* gene sequence, we assessed the ability of AGG/CCT interruptions to modulate both the ability of the CGG/CCG repeat DNA to incorporate into a nucleosome and the position of the repeat DNA around the histone core, and thus define the influence of AGG/CCT interruptions on the functional organization of the *FMR1* gene. By understanding the interaction between the interrupted and uninterrupted repeats and the histone core, we can evaluate the innate behaviors underlying the organization of the CGG/CCG repeats in the *FMR1* gene.

## Results and discussion

### Nineteen CGG/CCG repeats have no effect on incorporation into nucleosomes

To assess the ability of different DNA substrates to form nucleosomes we performed competitive nucleosome incorporation assays in which the radiolabeled DNA of interest along with unlabeled competitor, calf-thymus DNA, is exchanged onto the histone core of nucleosomes isolated from chicken erythrocytes [39-41]. Native PAGE is then used to separate radiolabeled DNA that is free and did not incorporate into a nucleosome from DNA that incorporated into a nucleosome.

**Figure 1:**
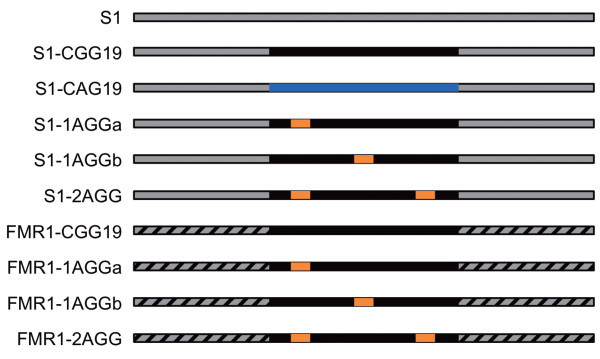
**Schematic representations of the DNA used in this study.** S1 serves as a control and contains no triplet repeat tracts. S1-CGG19 contains 19 CGG/CCG repeats (black) within the S1 flanking sequence (gray). S1-CAG19 contains 19 CAG/CTG repeats (blue) within the S1 flanking sequence (gray). S1-1AGGa, S1-1AGGb, and S1-2AGG contain AGG/CCT interruptions (orange) within the repeat tract. FMR1-CCG19, FMR1-1AGGa, FMR1-1AGGb, and FMR1-2AGG also contain CGG/CCG repeats (black) and AGG/CCT interruptions (orange), however they contain the *FMR1* flanking sequence (NG_007529.1, dashed). Please refer to the supporting information for the DNA sequences.

Before we can investigate the ability of AGG/CCT interruptions to modify the behavior of CGG/CCG repeats in a nucleosome, we must first understand the behavior of uninterrupted repeats. To assess the innate ability of healthy-length CGG/CCG repeats to incorporate into nucleosomes, we designed a 146 base pair (bp) substrate containing 19 CGG/CCG repeats centered within the nucleosome positioning sequence S1 (S1-CGG19) (Figure 1). The complete S1 sequence is known to form a homogenous population of nucleosomes, where the DNA occupies the same rotational and translation position around the histone core [42,43]. We chose to use 19 CGG/CCG repeats as we have previously characterized the non-canonical structures adopted by the (CGG)_19_ oligonucleotide [28]. While the length of the DNA substrates might restrict nucleosome formation, we chose to limit the length to 146 bp, the length of DNA wrapped around a single histone core, to minimize the probability that these substrates would form a heterogeneous population of nucleosomes.

**Figure 2:**
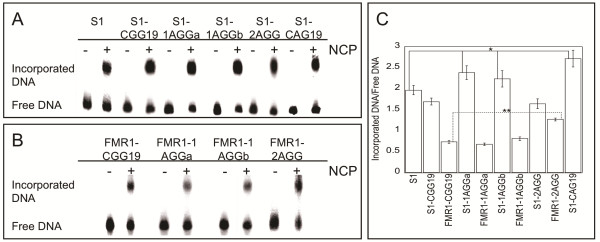
**Representative competitive nucleosome incorporation reactions.** To analyze the ability of each substrate to incorporate into a nucleosome, competitive nucleosome incorporation assays were performed under equilibrium conditions and the products of the reaction were resolved by native PAGE. An incorporation performed in the absence of chicken nucleosomes (NCP) (−NCP) and an incorporation performed in the presence of NCP (+NCP) are shown. The incorporation assays with the S1 series of substrates are shown in panel **A** and the incorporation assays with FMR1 series of substrates are shown in panel **B**. By quantifying the amount of radioactivity in both the incorporated DNA and free DNA bands, the ratio of incorporated DNA to free DNA was calculated for each substrate and is shown in panel **C**. The error bars represent the standard error for each substrate. Data represent a total of three biological replicates per substrate, each consisting of three technical replicates, for nine total values. The standard used for statistical comparison of the S1 series was the S1 substrate while the FMR1-CGG19 was used as the standard for statistical comparison of the FMR1 series. *p ≤ 0.05, **p ≤ 0.005 by Student’s T-Test.

The S1-CGG19 DNA, along with a 146 bp S1 control that lacks triplet repeats (S1), were used in a competitive nucleosome incorporation assay. In this assay, the radiolabeled DNA of interest along with unlabeled competitor, calf-thymus DNA, is exchanged onto the histone core of nucleosomes isolated from chicken erythrocytes. Native PAGE is then used to separate radiolabeled DNA that is free and did not incorporate into a nucleosome from DNA that incorporated into a nucleosome. When 19 CGG/CCG repeats are centered in the S1 sequence (S1-CGG19) there is no significant change in the ratio of incorporated DNA to free DNA (Figure 2A, 2C), relative to the S1 control. This contrasts with the observed increase for S1-CAG19, where 19 CAG/CTG repeats are centered in the S1 sequence (Figure 2A, 2C); notably, it is a CAG/CTG repeat sequence that expands to cause Huntington’s Disease, Myotonic Dystrophy, and Spinocerebellar Ataxias [7,8,44-47]. To our knowledge, this is the first direct comparison of these triplet repeat sequences where the number of repeats, flanking sequence, and overall DNA length are identical. Therefore, the 1.8 fold difference in nucleosome incorporation observed for S1-CGG19 and S1-CAG19 is derived exclusively from the identity of the triplet repeat sequence and provides a quantitative measure of the differing ability of these repeats to form nucleosomes under these conditions.

In previous experiments designed to observe the location of nucleosome formation on a closed, circular plasmid by electron microscopy, 76 CGG/CCG repeats were found to reduce nucleosome formation, meaning that the region of the plasmid containing the CGG/CCG repeats was populated with a nucleosome less frequently than other regions of the plasmid [35]. In other experiments, 10 and 24 CGG/CCG repeats placed in the center of a pUC19 fragment incorporated into a nucleosome less readily than a control that lacked the repeats. However, it is notable that these experiments were performed using DNA substrates that did not contain uniform flanking sequences, opening the possibility that the primary sequence of the flanking DNA could be affecting the observed results. Furthermore, it is also possible that rather than the CGG/CCG repeats themselves decreasing nucleosome incorporation, the introduction of the repeats disrupted a region of the pUC19 DNA sequence that promotes nucleosome incorporation. In other work, experiments performed with 6 CGG/CCG repeats in the histone H4 gene, found that CGG/CCG repeats can favorably contribute to nucleosome incorporation, though these experiments were also performed without regard for flanking sequence [37]. Perhaps our experiments with 19 repeats represent a middle ground where the length of the CGG/CCG repeat tract is too long to facilitate nucleosome incorporation but is not long enough to destabilize interaction with the histone core, at least within this context.

### A single AGG/CCT interruption increases nucleosome incorporation of a CGG/CCG repeat tract

To investigate the consequences of interruptions on nucleosome incorporation, we introduced AGG/CCT interruptions into the S1-CGG19 duplex (Figure 1). We placed the interruptions in the same locations as in previously investigated (CGG)_19_ oligonucleotides, where the interruptions were found to alter the non-canonical structures formed and to decrease the thermodynamic stability [28]. When a single CGG/CCG is replaced with AGG/CCT in either the fifth or ninth repeat (S1-1AGGa and S1-1AGGb, respectively) there is a ~1.5-fold increase in the incorporation ratio as compared to S1-CGG19 (Figure 2A and C). This result indicates that the presence of just one AGG/CCT interruption significantly impacts the ability of the DNA to interact with the histone core.

It is noteworthy that this observed increase in nucleosome incorporation is due to a change in 1 out of 146 bps; to introduce the AGG/CCT interruption, a C/G bp was changed to A/T. Based on analysis of the free energy (ΔG) of DNA bending for these (Additional file 1: Figure S1) and other substrates [34,48-50], it has traditionally been proposed that because AGG/CCT interruptions represent a localized decrease in ΔG within the *FMR1* repeat tract, thereby decreasing the energetic barrier for CGG/CCG repeat bending around the histone core, they make the incorporation of the interrupted sequences into a nucleosome slightly more energetically favorable. The inclusion of an AGG/CCT interruption also alters the calculated stiffness of the CGG/CCG repeat tract (Additional file 1: Figure S2). However, it is likely that other structural factors not accounted for in these calculations, such as the ability to compress the minor groove and accommodate the unique constraints imposed by bending around the histone core, also play a role in determining the affinity for the histone core. Indeed, differences in DNA stiffness alone does not account for the increased incorporation of the S1 DNA containing CAG/CTG repeats, indicative of the many factors that influence nucleosome formation. As discussed in our earlier work, the affinity of CAG/CTG-containing DNA likely comes from formation of a helical periodicity congruent with that of the region surrounding the dyad axis; it is possible that the S1-1AGGa and S1-1AGGb substrates also adopt a periodicity congruent with this region [39].

Interestingly, when an AGG/CCT interruption is included at both the fifth and fifteenth repeats (S1-2AGG), the level of incorporation is comparable to the uninterrupted S1-CGG19 (Figure 2A and C). We had anticipated that DNA containing two interruptions would incorporate more readily than S1-CGG19, as observed for the sequences with a single interruption. We speculate that the lack of increased incorporation observed for the S1-2AGG substrate is due to the position of the interruptions within the repeat tract. In the S1-2AGG substrate, the interruptions are positioned equidistant from the ends of the repeat tract, with ten intervening CGG/CCG repeats located in the center of the DNA. Because of the positioning effects of the S1 flanking sequence, this configuration places an interruption near either edge of the dyad axis, a region within the nucleosome where the DNA is locally underwound [38,51,52]. The intervening CGG/CCG repeats may be constrained by the underwinding associated with this region, leading to lower incorporation. Notably, the results obtained with one and two AGG/CCT interruptions underscore the importance of both the location of the interruption within the repeat tract and the location of DNA around the histone core.

The relationship between nucleosome incorporation and the presence of AGG/CCT interruptions in CGG/CCG repeat tracts has been investigated previously [34]. Using DNA fragments derived from human *FMR1* repeat tracts, when 3 or 2 interruptions were present in tracts of 39 or 53 CGG/CCG repeats respectively, the interruptions did not influence nucleosome incorporation on histones isolated from HeLa cells. However, the presence of the interruptions did increase formation of hyperacetylated nucleosomes. While we observe an increase in incorporation when a single interruption is present, the histones used in our experiments are isolated from chicken erythrocytes and will likely contain very low levels of acetylation as the genetic material is stored as heterochromatin within the erythrocyte, but the absolute measure of epigenetic modifications is unknown. Interestingly, when ATG/CAT interruptions were added to a CAG/CTG repeat tract, the incorporation ratio decreased [34].

### The FMR1 gene flanking sequence decreases nucleosome incorporation

Using the S1 series of substrates, it is possible to determine the innate ability the AGG/CCT interruptions to modulate CGG/CCG repeat incorporation into a nucleosome. However, it is equally important to understand the effect of interruptions when the CGG/CCG repeats are flanked by the biologically-relevant *FMR1* gene sequence. Thus, we designed DNA substrates that have the same repeat and interruption configuration as those in the S1 series, but contain the *FMR1* gene flanking sequence (Figure 1). In all FMR1 substrates, the observed level of incorporation decreases, relative to the corresponding S1 substrate (Figure 2B and C). This decrease in incorporation is likely due at least in part to an increase in the ΔG of bending for the *FMR1* gene flanking sequence relative to the S1 flanking sequence (Additional file 1: Figure S1). Interestingly, while a single AGG/CCT interruption yields an increase in incorporation of the S1 substrates, there is no significant change when a single AGG/CCT interruption is present in the FMR1 substrates. In contrast, while the two AGG/CCT interruptions in S1-2AGG produced no change in the amount of incorporation relative to the S1-CGG19, there is a significant increase in the incorporation observed in the FMR1-2AGG substrate relative to the FMR1-CGG19 substrate. Thus, not only are the number and placement of the AGG/CCT interruptions important factors in determining the ability of CGG/CCG repeats to incorporate into a nucleosome, but the flanking sequence is also important, as it can further modulate the impact of the interruptions.

In the cells of FXS patients, disease length CGG/CCG repeats are associated with heterochromatic markers, indicating that these repeats are contained within tightly packed heterochromatin [14-16,32,33,53]. Based on nucleosome incorporation assays, this silencing does not come from the innate ability of CGG/CCG repeats or the *FMR1* flanking sequence to form nucleosomes; both the CGG/CCG repeats and *FMR1* flanking sequence decrease the amount of DNA incorporated into a nucleosome, relative to their respective controls. Therefore, it is most likely that heterochromatic markers associated with the expanded CGG/CCG repeats silence the *FMR1* gene [16,34,36,37]. Importantly, as heterochromatin formation appears to happen through trans-acting factors and not an innate affinity for the histone core by the CGG/CCG repeat and FMR1 flanking sequence, heterochromatin formation may be reversed by targeting the correct trans-acting factor.

### Exonuclease III digestion reveals the position of the DNA around the histone core

**Figure 3:**
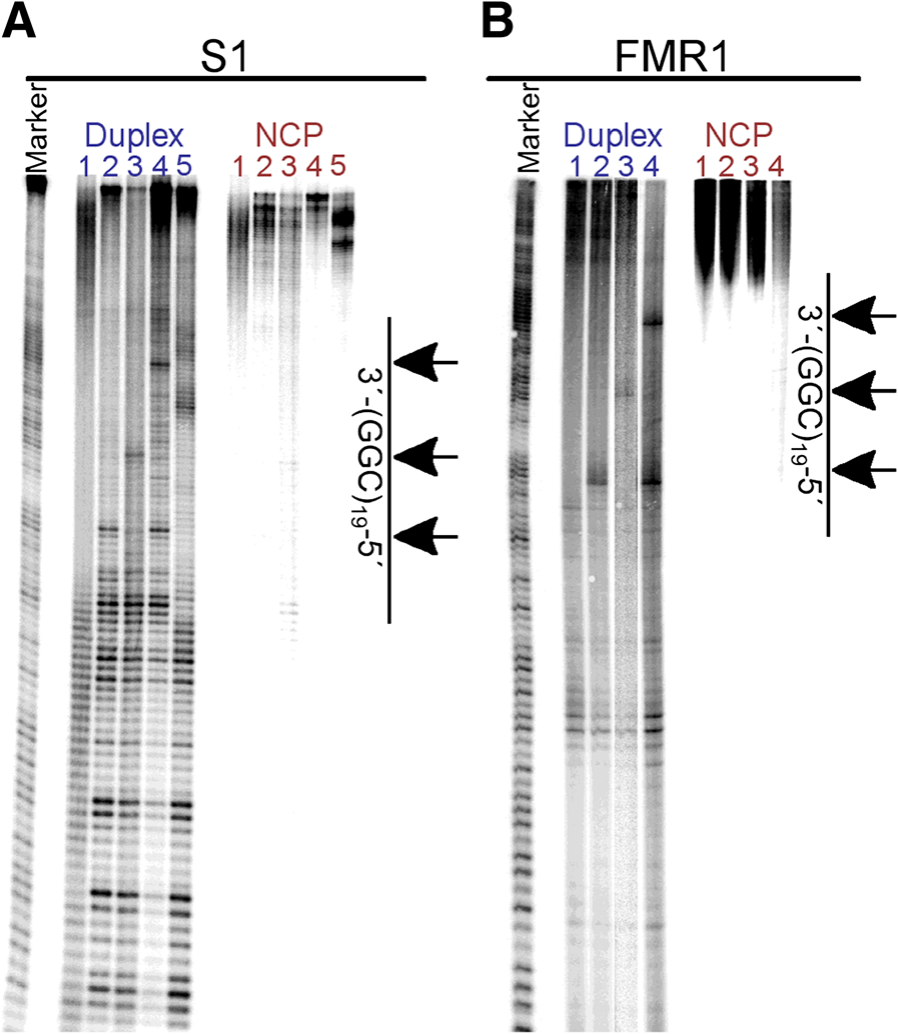
**Exonuclease III digestion reveals weaker interactions between the ends of the DNA and the histone core.** Exo III digestion of the CGG-containing strands of the S1 substrates are shown in panel **A**, while digestion of the CGG-containing strands of the FMR1 substrates are shown in panel **B**. Reactions were performed on both free duplex (labeled in blue) and nucleosome (NCP, labeled in red) substrates. In panel **A**, lanes 1 contain the S1 control, lanes 2 contain S1-CGG19, lanes 3 contain S1-1AGGa, lanes 4 contain S1-1AGGb, and lanes 5 contain S1-2AGG. In panel **B**, lanes 1 contain FMR1-CGG19, lanes 2 contain FMR1-1AGGa, lanes 3 contain FMR1-1AGGb, and lanes 4 contain FMR1-2AGG. The marker lane consists of the Maxam-Gilbert A/G sequencing reaction performed on S1a. The location of the repeat tract and the A of the interruptions (arrows) are indicated on the right-hand side of the gel.

We have previously used Exonuclease III (Exo III), a 3′ to 5′ exonuclease, to investigate the general positioning of the DNA duplex around the histone core in well-positioned CAG/CTG repeat-containing substrates [39]. We have applied that same technique to investigate the interaction between the CGG/CCG repeat-containing DNA and the histone core. In separate experiments either the CGG- or CCG-containing strand was 5′-radiolabeled, which allows visualization of reactivity at both ends of the duplex. Both free duplex and nucleosome substrates were subjected to partial digestion by Exo III and the products were resolved by denaturing PAGE. As Exo III digests the DNA duplex in the nucleosome, the enzyme pauses where the DNA duplex is in contact with the histone core, causing high levels of strand cleavage [54-56]. When the CGG/CCG repeats, both with and without AGG/CCT interruptions, are contained within the S1 positioning sequence, there is clear reactivity along the length of each free duplex, evidenced by the appearance of discrete bands in the denaturing gel (Figure 3A, duplex). However, once the DNA is incorporated into a nucleosome, there is a change in the reactivity; namely strand cleavage only arises from Exo III digestion of the DNA at the end of the duplex (Figure 3A, NCP, top of the gel). The same change in reactivity is present at the same location in the FMR1 substrates as well (Figure 3B, NCP, top of the gel). It is interesting to note that, while reactivity in both the S1 and FMR1 nucleosomal substrates is restricted to the same region, the reactivity within that region is different. In the S1 substrates there are discreet, high intensity regions corresponding to the Exo III pause sites. However there are no distinct pause sites present in the FMR1 substrates; the entire region is equally reactive. The difference in reactivity at the ends of the DNA indicates that the FMR1 DNA may not be uniformly positioned (*vide infra*).

### Interrupted and uninterrupted CGG/CCG repeats form canonical duplex in the nucleosome

We previously reported that the secondary CCG/CGG repeat region found in the *huntingtin* (*HTT*) gene is reactive with S1 nuclease upon incorporation into a nucleosome, indicating the presence of a single stranded region, a region which we referred to as kinked [39]. We performed the same experiments using the S1- and FMR1-CGG DNA to investigate whether CGG/CCG repeats also form kinks in this context (Additional file 1: Figure S3). We find no areas of increased reactivity to S1 nuclease in nucleosome samples containing these CGG/CCG repeats (compared to the free duplex samples), indicating that they behave as canonical duplex when incorporated into the nucleosome. Notably, the CCG/CGG repeats in the HTT substrate were located at the 3′ end of the 146 bp duplex, while the CGG/CCG repeats in these substrates are in the center of the duplex. The difference in position within the DNA substrate will place the repeats in different environments within the nucleosome, possibly explaining the difference in behavior.

### CGG/CCG repeats reposition DNA in the nucleosome; AGG/CCT interruptions further alter the positioning

**Figure 4:**
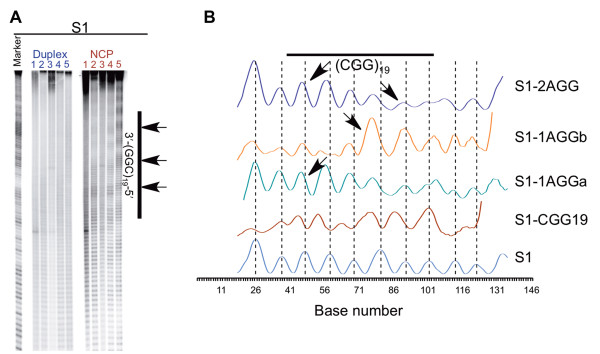
**Hydroxyl radical footprinting reveals the periodicity of the DNA around the histone core.** Radiolabeled samples, both free duplex (labeled in blue) and nucleosomes (NCP, labeled in red) substrates, were exposed to hydroxyl radicals, revealing a characteristic pattern of oscillating high and low reactivity as the DNA wraps around the histone core. Reactivity toward hydroxyl radical for the CGG-containing strands of the S1 substrates are shown in panel **A**. Reactions were performed on both free duplex (labeled in blue) and nucleosome (NCP, labeled in red) substrates. In panel **A**, lanes 1 contain the S1 control, lanes 2 contain S1-CGG19, lanes 3 contain S1-1AGGa, lanes 4 contain S1-1AGGb, and lanes 5 contain S1-2AGG. The marker lane consists of the Maxam-Gilbert A/G sequencing reaction performed on S1a. The location of the repeat tract and interruptions are indicated on the right-hand side of the gel. Panel **B** shows the reactivity at each nucleotide, generated from the gel presented in **A**. The dashed lines indicate the maxima of the S1 substrate and the arrows indicate the position of the A of the AGG interruptions.

To further understand how the AGG/CCT interruptions modify the behavior of the CGG/CCG repeat tract in the nucleosome, we used hydroxyl radical footprinting to determine the position of the DNA relative to the histone core. The hydroxyl radical abstracts the C5′ hydrogen atom of the sugar-phosphate backbone, located in the minor groove, inducing strand breaks along the length of the DNA backbone [42,51,57-59]. First, we used this technique to analyze the ability of 19 CGG/CCG repeats to reposition the S1 DNA in a nucleosome. Separate experiments were performed with either the CGG-containing (Figure 4) or CCG-containing strand (Additional file 1: Figure S4) radiolabeled, and the products were resolved by denaturing PAGE. Regions of most intense strand cleavage indicate where the minor groove is solvent exposed. Thus, exposure of free duplex to hydroxyl radicals results in cleavage along the entire DNA substrate in a relatively even manner, observed as the appearance of discreet bands in the gel (Figure 4A, Additional file 1: Figure S4A; duplex). However, once the DNA is incorporated into a nucleosome, the cleavage pattern changes to the characteristic pattern of oscillating high and low reactivity associated with nucleosome incorporation (Figure 4A, Additional file 1: Figure S4A; NCP). This oscillation results from the wrapping of the DNA around the histone core. In areas where the minor groove faces away from the histone core, cleavage is high, but in areas where the minor groove faces in toward the histone core, cleavage is low. The amount of cleavage at each nucleotide was determined and used to construct the plots shown in Figure 4B and Additional file 1: Figure S4B, allowing comparison of the overall DNA position between the various DNA substrates. The characteristic oscillating pattern produced by reaction of the S1 nucleosome substrates with the hydroxyl radical indicates a uniform population of nucleosomes, where the DNA occupies a single rotational and translational position. The vertical, dashed lines in Figure 4B and Additional file 1: Figure S4B indicate the maxima for the S1 control. It is from these lines that any changes in the overall position of the DNA are observed. When 19 CGG/CCG repeats are located in the center of the S1 DNA, there is a shift of ~4 bases in the position of the maxima and minima relative to the S1 substrate, particularly in the repeat region, indicating a difference in how the DNA is positioned in the nucleosome. When an AGG/CCT interruption replaces a CGG/CCG repeat, the maxima and minima are again offset, signifying that the DNA again positions differently, now occupying a different overall position than either the S1 or S1-CGG19 nucleosomes.

The hydroxyl radical footprinting data discussed above can be broken down into two positioning components: the rotational and translational positioning. To examine the rotational positioning of each DNA substrate, the footprinting data of the two complementary strands is overlaid and the location of the minor groove determined by locating the area between a maximum in one strand and the nearest maximum in the complementary strand, shaded in gray in Additional file 1: Figure S5 [42]. By examining the position of the minor groove, the rotational positioning of different substrates can be compared. Upon addition of 19 CGG/CCG repeats to the S1 DNA, there is a shift in the rotational positioning of the DNA in the nucleosome. Healthy-length CAG/CTG repeats have also been shown to alter the rotational positioning of the S1 DNA in the nucleosome [39]. Interestingly, upon the addition of the AGG/CCT interruptions, the S1-1AGGa, S1-1AGGb, and S1-2AGG DNA all adopt the same rotational position, different from both the S1 control and the S1-CGG19 substrates. Thus, not only does the presence of the CGG/CCG repeat alter the rotational positioning of DNA in the nucleosome, but the addition of an AGG/CCT interruption, a change of 1 or 2 bps out of 146, further alters the rotational positioning.

**Figure 5:**
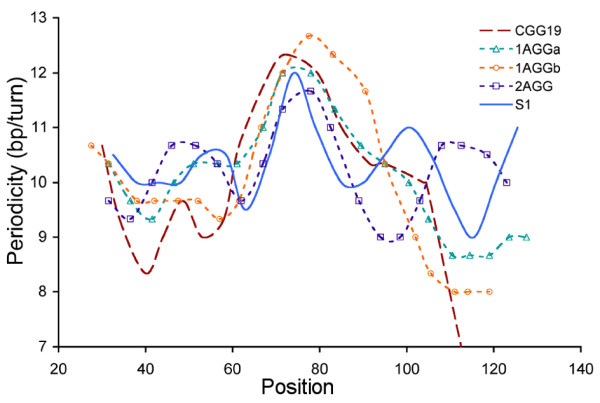
**Translational positioning curves reveal a change in the position of the dyad axis for the S1 series.** Translational positioning curves were created by determining the location of and distance between each consecutive maxima and minima in the hydroxyl radical footprinting data. The locations of the maxima and minima are then graphed with respect to their periodicity. The curve reveals the variation in periodicity around the histone core, and the highest peak indicates the location of the dyad axis. In the S1 series, the translational positioning curves reveal 2–5 base pair offsets in the location of the dyad axis.

By mapping the periodicity between each maxima and minima in the CGG-containing strand (Figure 5), the translational positioning and, importantly, the nucleotide positioned at the dyad axis were determined for each S1 substrate (Table 1) [42]. The nucleotide positioned at the dyad is the most accessible measure of the translational positioning; if substrates have the same nucleotide positioned at the dyad they have the same general translational position. When the CGG/CCG repeat tract replaces the central region of the S1 sequence, there is a 2 bp shift in the position of the dyad axis. Healthy-length CAG/CTG repeats also alter the translational positioning of DNA in the nucleosome [39]. Upon examination of the translational positioning curves, it is apparent that each of the interrupted substrates, S1-1AGGa, S1-1AGGb, and S1-2AGG, has a different dyad axis position than S1-CGG19. Thus, as we observed for the rotational positioning, the CGG/CCG repeat alters the translational positioning relative to the S1 control, and the addition of an interruption further alters the translational positioning.

**Table 1:** Position of the Dyad and translational shift

**Substrate**	**Dyad position**	**Translational shift**
		**(base pairs**^**a**^**)**
S1	74	-
S1-CGG19	72	−2
S1-1AGGa	74.75	+0.75
S1-1AGGb	77.5	+3.5
S1-2AGG	78	+4

^a^With respect to the S1 control.

The ability of AGG/CCT interruptions to alter the positioning of the CGG/CCG repeat may contribute to the “gray zone” between healthy and pre-mutation lengths, where uninterrupted repeats classified as healthy-length can expand, but longer, interrupted repeats do not [23,25-27]. Perhaps by modulating either the ability of the repeat tract to form a nucleosome or by altering its rotational and translational position around the histone core, the interruptions offer some protection from expansion. Furthermore, as a single interruption has an innate ability to reposition the repeat tract in a nucleosome, and since the loss of a single interruption has been linked with expansion [23,25-27], the loss of the positioning effect of the AGG/CCT interruption may enhance the expansion process.

### CGG/CCG repeats decrease the periodicity of DNA in the nucleosome, but an AGG/CCT interruption increases periodicity

To determine the periodicity, or number of bps per helical turn of DNA, associated with either the entire DNA duplex or the periodicity associated with just the repeat region, a sine wave was fit to each curve presented in Figure 4B. The periodicities are given in Table 2 and Additional file 1: Table S2. Consistent with previous experiments from our [39] and other [42] labs, the periodicity of the S1 control is 10.37 bp/turn. Upon inclusion of the 19 CGG/CCG repeats, the periodicity decreases to 10.14 bp/turn. However, when the AGG/CCT interruptions are present, the periodicity returns to ~10.4 bp/turn. The same pattern is observed when the periodicity is determined specifically for the repeat region; the repeat region of the S1-CGG19 substrate has a lower periodicity than the repeat region of any of the substrates containing interruptions.

**Table 2:** Base pairs per helical turn for S1 nucleosomal substrates

**Substrate**	**Total**	**Repeat region**
S1a	10.37 ± 0.01	N/A
S1-CGG19	10.14 ± 0.05	10.38 ± 0.03
S1-1AGGa	10.36 ± 0.02	10.56 ± 0.03
S1-1AGGb	10.40 ± 0.04	10.55 ± 0.04
S1-2AGG	10.40 ± 0.02	10.49 ± 0.03

The periodicity of both the uninterrupted and interrupted healthy-length CGG/CCG repeat tracts is less than that of healthy-length CAG/CTG repeats, which is consistently ~10.7 bp/turn [39]. However, both repeats are located in the same area of the nucleosome: the dyad axis, an area that generally adopts a periodicity of ~10.7 bp/turn, which is best for bending around that region of the histone core. The difference in periodicity between the CGG/CCG and CAG/CTG repeats provides an additional rationale for the differences observed in DNA incorporation for these two triplet repeat sequences. While the CAG/CTG repeats adopt a periodicity well suited for bending around the dyad axis, uninterrupted CGG/CCG repeat tracts cannot adopt such a relatively large periodicity. However, once an interruption is present, the repeat tract can now adopt a periodicity closer to the 10.7 bp/turn preferred for bending around the dyad axis, leading to higher levels of incorporation.

Not only does the periodicity determined for the CGG/CCG repeats inform the ability of the repeats to incorporate into the nucleosome, it may also explain why the CGG/CCG repeats positioned at the dyad axis remain as canonical duplex while CGG/CCG repeats positioned away from the dyad axis are kinked. The kinked CCG/CGG repeats in the *HTT* gene are in a region that is locally overwound; the periodicity is only ~10 bp/turn and kinking may relieve this overwinding [39]. The CGG/CCG repeats in this study are not overwound, but rather are underwound.

### FMR1 substrates do not form nucleosomes with a single DNA position

**Figure 6:**
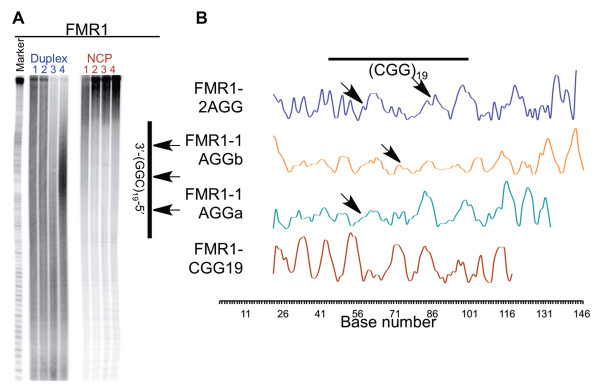
**Hydroxyl radical footprinting reveals the periodicity of the DNA around the histone core.** Radiolabeled samples, both free duplex (labeled in blue) and nucleosomes (NCP, labeled in red) substrates, were exposed to hydroxyl radicals. Reactivity toward hydroxyl radical for the CGG-containing strands of the FMR1 substrates are shown in panel **A**. Reactions were performed on both free duplex (labeled in blue) and nucleosome (NCP, labeled in red) substrates. In panel **A**, lanes 1 contain FMR1-CGG19, lanes 2 contain FMR1-1AGGa, lanes 3 contain FMR1-1AGGb, and lanes 4 contain FMR1-2AGG. The marker lane consists of the Maxam-Gilbert A/G sequencing reaction performed on S1a. The location of the repeat tract and interruptions are indicated on the right-hand side of the gel. Panel **B** shows the reactivity at each nucleotide, generated from the gel presented in **A**. The arrows indicate the position of the **A** in the AGG interruptions.

In contrast to the hydroxyl radical footprint observed for the S1 substrates, the FMR1 substrates produce ambiguous results (Figure 6 and Additional file 1: Figure S6). While reaction of the free duplex DNA with hydroxyl radicals produces even reactivity along the DNA substrate, just as observed in the S1 substrates, the oscillation between high and low reactivity when the FMR1 DNA is incorporated into a nucleosome is different from that observed for the S1 substrates (compare Figure 4B with Figure 6B). This cleavage pattern indicates that the FMR1 substrates form heterogeneous populations of nucleosomes where the DNA adopts several different positions around the histone core, leading to overlapping oscillating patterns. Interestingly, the footprint of the FMR1-CGG19 nucleosome does have an oscillation more similar to those seen in the S1 DNA, indicating that there may be only a few very closely related DNA positions. However, the footprints of the interrupted substrates look quite different from that of the FMR1-CGG19, indicating that they may adopt many different DNA positions. The ability of the FMR1 substrates to adopt multiple positions relative to the histone core indicates that the repeat tract is less limited in its positioning options as compared to the S1 substrates. This may explain why the FMR1-2AGG substrate has the highest incorporation of the FMR1 series; because the FMR1 flanking sequence cannot dictate the position of the DNA as effectively as the S1 flanking sequence, the DNA is better able to balance the competing positioning forces of the repeat tract, the AGG/CCT interruptions, and the FMR1 flanking sequence, leading to higher levels of incorporation. Furthermore, the ability of the interrupted FMR1 substrates to adopt several equivalent DNA positions may indicate that they are more easily remodeled, allowing proper progression of the replication machinery; while CGG/CCG repeats have been shown to promote polymerase stalling in a bacterial plasmid [60], they do not promote polymerase stalling in eukaryotic cells [61,62]. Unfortunately, detailed analysis of the hydroxyl radical footprinting data requires a homogenous population, thus we cannot draw any specific conclusions about how the *FMR1* gene sequence might modulate the position of the interrupted and uninterrupted CGG/CCG repeat tracts.

The formation of a heterogeneous population by the FMR1 substrates was unexpected, considering we designed our DNA substrates with only 146 bps to minimize the possibility of just such an occurrence. However, CGG/CCG repeats in a larger fragment of the human *FMR1* gene have previously been shown to form multiple translational positions, and the sequence flanking the repeats has also been shown to alter nucleosome positioning *in vivo*[37,63]. It is important to consider that the AGG/CCT interruptions are likely altering the rotational and translational positions of the FMR1 substrates in a similar manner as in the S1 substrates, but because of the formation of multiple species of nucleosomes, those changes cannot be detected by this technique.

## Conclusions

Here, we investigated not only the incorporation and positioning ability of an uninterrupted CGG/CCG repeat tract in the nucleosome, but also the ability of AGG/CCT interruptions to alter those properties. We find that, while 19 CGG/CCG repeats themselves have no impact on incorporation of the 146 bp S1 duplex, a single AGG/CCT interruption significantly increases incorporation into the nucleosome. When the S1 flanking sequence is replaced by the *FMR1* gene sequence, the changes induced by the addition of AGG/CCT interruptions are much smaller, primarily due to the overall barrier to incorporation presented by the FMR1 flanking sequence. However that barrier is partially over come by the presence of two AGG/CCT interruptions, which shows a significant increase in nucleosome formation compared to the uninterrupted substrate. When flanked by the S1 sequence, not only does the presence of interruptions alter the ability of the repeat tract to form a nucleosome, but once incorporated, interruptions also alter the rotational and translational positioning of the repeat tract around the histone core, as well as the periodicity of the DNA. However, the same repeats flanked by the FMR1 sequence form a heterogeneous population of nucleosomes.

It has been proposed that AGG/CCT interruptions play a protective role within the CGG/CCG repeat tract by destabilizing non-canonical secondary structures [28,29]. The work presented here provides insight into an additional protective function: the presence of AGG/CCT interruptions may alter the behavior of the repeat tract in the packaged genome. The ability to alter the behavior of the repeat tract in the nucleosome may partially explain the difference in expansion ability between an interrupted and uninterrupted repeat tract whose length falls within the so called “gray zone” of healthy-length repeats, and why the loss of a single interruption, particularly at the 3′ end of the repeat, can have such dire consequences. Combining their ability to destabilize non-canonical secondary structures and their ability to alter the behavior of the repeat tract within the packaged genome, the AGG/CCT interruptions clearly play an important role in maintenance of the CGG/CCG repeat tact in the *FMR1* gene.

## Methods

### Oligonucleotide synthesis and purification

Oligonucleotides were synthesized using standard phosphoramidite chemistry and purified by HPLC and gel purification as previously described (sequences can be found in Additional file 1: Table S1) [39].

### Competitive nucleosome incorporation assay

Oligonucleotides were 5′-^32^P end-labeled using T4 polynucleotide kinase (New England Biolabs) following the manufacturer’s protocol. To form duplex, radiolabeled DNA, along with a 1.5-fold excess of its complement, was resuspended to a final volume of 60 μL in Tris buffer (10 mM Tris–HCl, 100 mM NaCl, pH 7.5), heated at 95°C for 5 min and cooled to 25°C at a rate of 1°C/min.

Nucleosomes were isolated from chicken erythrocytes using previously published protocols [39,40]. Following previously published protocols [39,41], four competitive nucleosome incorporation reactions were prepared per DNA substrate; three reactions contained the radiolabeled DNA duplex and nucleosomes while one reaction contained radiolabeled DNA duplex but lacked nucleosomes. The incorporation reactions were completed three times for each DNA sequence yielding a total of nine incorporations per DNA.

### Exonuclease III, S1 nuclease, and hydroxyl radical footprinting reactions

For enzymatic or chemical probing, DNA was exchanged into the nucleosomes and the reactions on nucleosomal substrates and free duplex were carried out as previously described [39]. Briefly, nucleosome exchange was performed, and the sample subjected to digestion. After stopping the reaction with either EDTA (Exo III and S1 nuclease) or 50% glycerol (hydroxyl radical footprinting), samples were applied to a 5% native polyacrylamide gel. Incorporated DNA was excised from the gel and the DNA recovered. Reactions were also carried out of free duplex. Both the free duplex and incorporated samples were electrophoresed on a 8% denaturing polyacrylamide gel. A standard Maxam-Gilbert A/G reaction performed on either strand of S1 (S1a or S1b, Additional file 1: Table S1) was used to create a marker.

### Image processing

Phosphorplates were imaged using a BioRad Pharos scanner and the associated Quantity One software. Image processing was done using BioRad’s Image Lab software and Origin 8.2 (OriginLab Corporation), as described previously [39].

### Availability of supporting data

All supporting data are included in the additional file included with this article.

## Abbreviations

bp: Base pair; DMT: Dimethoxytrityl; EDTA: Ethylenediaminetetraacetic acid; EB: Exchange buffer; Exo III: Exonuclease III; FXS: Fragile X syndrome; FMR1: Fragile X mental retardation 1; HTT: Huntingtin; NCP: Nucleosome core particle; PAGE: Polyacrylamide gel electrophoresis; TEAA: Triethyl ammonium acetate.

## Competing interests

The authors declare that they have no competing interests.

## Authors’ contributions

CBV and SD designed research, CBV carried out research, and CBV and SD wrote the paper. Both authors read and approved the final manuscript.

## Supplementary Material

Additional file 1: Table S1 Sequences of DNA used in these experiments. **Table S2.** Nucleotides Per Helical Turn for S1 CCG-containing Nucleosomal Substrates. **Figure S1.** ΔG of bending values calculated for the S1 and FMR1 substrates. **Figure S2.** Stiffness curves for S1 and FMR1 substrates. **Figure S3.** S1 nuclease digestion of the S1 and FMR1 DNA substrates. **Figure S4.** Hydroxyl radical cleavage of the CCG-containing S1 substrates. **FigureS5.** Rotational positioning curves for the S1 substrates. **Figure S6.** Hydroxyl radical cleavage of the CCG-containing FMR1 substrates. Click here for file
