# Supplementary material for: AGG/CCT interruptions affect nucleosome formation and positioning of healthy-length CGG/CCG triplet repeats
Source: BMC Biochem. 2013 Nov 22;14:33. doi: 10.1186/1471-2091-14-33 (PMC3870987; doi:10.1186/1471-2091-14-33)
Supplement: Additional file 1: Table S1 — Sequences of DNA used in these experiments. Table S2. Nucleotides Per Helical Turn for S1 CCG-containing Nucleosomal Substrates. Figure S1. ΔG of bending values calculated for the S1 and FMR1 substrates. Figure S2. Stiffness curves for S1 and FMR1 substrates. Figure S3. S1 nuclease digestion of the S1 and FMR1 DNA substrates. Figure S4. Hydroxyl radical cleavage of the CCG-containing S1 substrates. FigureS5. Rotational positioning curves for the S1 substrates. Figure S6. Hydroxyl radical cleavage of the CCG-containing FMR1 substrates. [file 1471-2091-14-33-S1.pdf]

**Supporting Information**

# AGG/CCT Interruptions Affect Nucleosome Formation and Positioning of Healthy-Length CGG/CCG Triplet Repeats

**Catherine B. Volle[1] and Sarah Delaney[2]**

[1] Department of Molecular Biology, Cell Biology and Biochemistry and [2] Department of Chemistry, Brown University, Providence, RI 02912, USA
Address correspondence to: Sarah Delaney, Brown University, 324 Brook Street, Providence RI 02912, USA. Email address: sarah_delaney@brown.edu. Telephone: 1 (401) 863-3590. Fax: 1 (401) 863-9368.

**Supporting Information Table S1:** Sequences of DNA used in these experiments.

**Supporting Information Table S2:** Nucleotides Per Helical Turn for S1 CCG-containing Nucleosomal Substrates.

**Supporting Information Figure S1:** ΔG of bending values calculated for the S1 and FMR1 substrates.

**Supporting Information Figure S2:** Stiffness curves for S1 and FMR1 substrates

**Supporting Information Figure S3:** S1 nuclease digestion of the S1 and FMR1 DNA substrates.

**Supporting Information Figure S4:** Hydroxyl radical cleavage of the CCG-containing S1 substrates.

**Supporting Information Figure S5:** Rotational positioning curves for the S1 substrates.

**Supporting Information Figure S6:** Hydroxyl radical cleavage of the CCG-containing FMR1 substrates.

## Tables:

Supporting Information Table S1: Primary sequence of DNA used in these studies

| Name | Sequence (5′- 3′) | Sequence (5′- 3′) |
|---|---|---|
| S1 | S1a:GTAAATTAATAGTTGAAGTTGTAGTAAATGTTAATGTAGATCTGTTGTTCCGATATTACCAAAACCTTCACCTAATAGCGCTAGTACACAGGAGAAGGACATGAACATGAACCTAATGAACACAACAAATAATGTAAGTGCCCATG | S1b:CATGGGCACTTACATTATTTGTTGTGTTCATTAGGTTCATGTTCATGTCCTTCTCCTGTGTACTAGCGCTATTAGGTGAAGGTTTTGGTAATATCGGAACAACAGATCTACATTAACATTTACTACAACTTCAACTATTAATTTAC |
| S1-CGG19 | GTAAATTAATAGTTGAAGTTGTAGTAAATGTTAATGTAGATCTGTT**(CGG)$_{19}$**AACATGAACCTAATGAACACAACAAATAATGTAAGTGCCCATG | CATGGGCACTTACATTATTTGTTGTGTTCATTAGGTTCATGTT**(CCG)$_{19}$**AACAGATCTACATTAACATTTACTACAACTTCAACTATTAATTTAC |
| S1-1AGGa | GTAAATTAATAGTTGAAGTTGTAGTAAATGTTAATGTAGATCTGTT**(CGG)$_4$AGG(CGG)$_{14}$**AACATGAACCTAATGAACACAACAAATAATGTAAGTGCCCATG | CATGGGCACTTACATTATTTGTTGTGTTCATTAGGTTCATGTT**(CCG)$_{14}$CCT(CCG)$_4$**AACAGATCTACATTAACATTTACTACAACTTCAACTATTAATTTAC |
| S1-1AGGb | GTAAATTAATAGTTGAAGTTGTAGTAAATGTTAATGTAGATCTGTT**(CGG)$_8$AGG(CGG)$_{10}$**AACATGAACCTAATGAACACAACAAATAATGTAAGTGCCCATG | CATGGGCACTTACATTATTTGTTGTGTTCATTAGGTTCATGTT**(CCG)$_{10}$CCT(CCG)$_8$**AACAGATCTACATTAACATTTACTACAACTTCAACTATTAATTTAC |
| S1-2AGG | GTAAATTAATAGTTGAAGTTGTAGTAAATGTTAATGTAGATCTGTT**(CGG)$_4$AGG(CGG)$_9$AGG(CGG)$_4$**AACATGAACCTAATGAACACAACAAATAATGTAAGTGCCCATG | CATGGGCACTTACATTATTTGTTGTGTTCATTAGGTTCATGTT**(CCG)$_4$CCT(CCG)$_9$CCT(CCG)$_4$**AACAGATCTACATTAACATTTACTACAACTTCAACTATTAATTTAC |
| FMR1-CGG19 | CGGCGGCGGTGACGGAGGCGCCGCTGCCAGGGGGCGTGCGGCAGCG**(CGG)$_{19}$**CTGGGCCTCGAGCGCCCGCAGCCCACCTCTCGGGGGCGGGCTC | GAGCCCGCCCCCGAGAGGTGGGCTGCGGGCGCTCGAGGCCCAG**(CCG)$_{19}$**CGCTGCCGCACGCCCCCTGGCAGCGGCGCCTCCGTCACCGCCGCCG |
| FMR1-1AGGa | CGGCGGCGGTGACGGAGGCGCCGCTGCCAGGGGGCGTGCGGCAGCG**(CGG)$_4$AGG(CGG)$_{14}$**CTGGGCCTCGAGCGCCCGCAGCCCACCTCTCGGGGGCGGGCTC | GAGCCCGCCCCCGAGAGGTGGGCTGCGGGCGCTCGAGGCCCAG**(CCG)$_{14}$CCT(CCG)$_4$**CGCTGCCGCACGCCCCCTGGCAGCGGCGCCTCCGTCACCGCCGCCG |
| FMR1-1AGGb | CGGCGGCGGTGACGGAGGCGCCGCTGCCAGGGGGCGTGCGGCAGCG**(CGG)$_8$AGG(CGG)$_{10}$**CTGGGCCTCGAGCGCCCGCAGCCCACCTCTCGGGGGCGGGCTC | GAGCCCGCCCCCGAGAGGTGGGCTGCGGGCGCTCGAGGCCCAG**(CCG)$_{10}$CCT(CCG)$_8$**CGCTGCCGCACGCCCCCTGGCAGCGGCGCCTCCGTCACCGCCGCCG |
| FMR1-2AGG | CGGCGGCGGTGACGGAGGCGCCGCTGCCAGGGGGCGTGCGGCAGCG**(CGG)$_4$AGG(CGG)$_9$AGG(CGG)$_4$**CTGGGCCTCGAGCGCCCGCAGCCCACCTCTCGGGGGCGGGCTC | GAGCCCGCCCCCGAGAGGTGGGCTGCGGGCGCTCGAGGCCCAG**(CCG)$_4$CCT(CCG)$_9$CCT(CCG)$_4$**CGCTGCCGCACGCCCCCTGGCAGCGGCGCCTCCGTCACCGCCGCCG |

Supporting Information Table S2: Nucleotides Per Helical Turn for S1 Nucleosomal Substrates

| Substrate | Total | Repeat |
|---|---|---|
| S1b | 10.37 ± 0.02 | N/A |
| S1-CCG19 | 10.23 ± 0.03 | 10.42 ± 0.02 |
| S1-1CCTa | 10.37 ± 0.02 | 10.52 ± 0.02 |
| S1-1CCTb | 10.41 ± 0.03 | 10.52 ± 0.02 |
| S1-2CCT | 10.41 ± 0.02 | 10.52 ± 0.02 |

**Figures:**

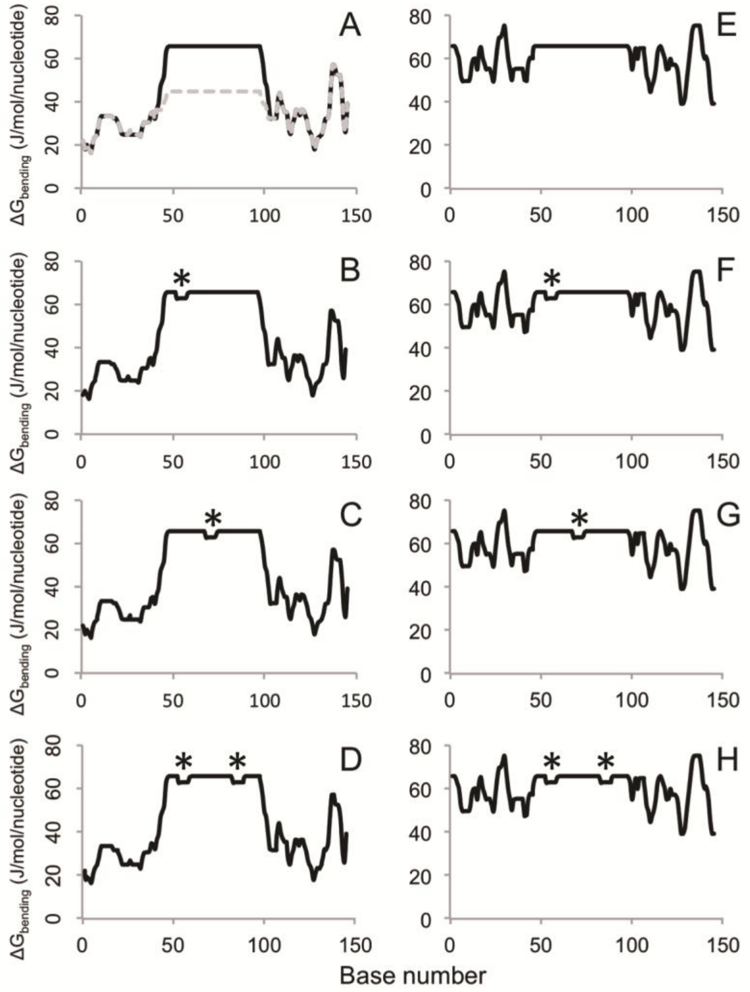


Supporting Information Figure S1: ΔG of bending values calculated for the S1 and FMR1 substrates. Using the equation described by Satchwell and Travers,[1] and as applied by Metzenberg,[2] the ΔG of bending was calculated across each substrate, using a 6-nucleotide window. S1-CGG19, S1-1AGGa, S1-1AGGb, and S1-2AGG (black) are shown in panels A-D respectively. S1-CAG19 (dashed gray) is also shown in panel A. FMR1-CGG19, FMR1-1AGGa, FMR1-1AGGb, and FMR1-2AGG (black) are shown in panels E-H respectively. The interruptions are indicated by *.

(1) Satchwell, S. C., and Travers, A. A. (1989) Asymmetry and polarity of nucleosomes in chicken erythrocyte chromatin, *The EMBO Journal 8*, 229–238.

(2) Metzenberg, S. (1996) On the formation of nucleosomes within the FMR1 trinucleotide repeat, *Am J Hum Genet 59*, 252–253.

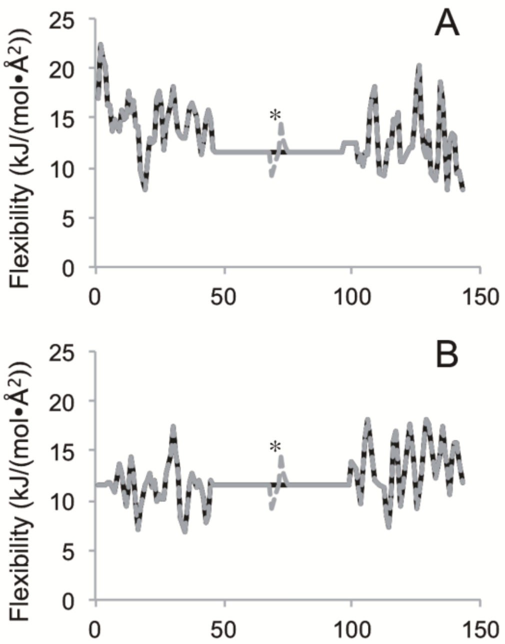


Supporting Information Figure S2: Stiffness curves calculated for several interrupted and uninterrupted substrates. Using the values described by Packer et al.,[3] we calculated the flexibility of the S1-CGG19 and S1-1AGGb (panel A) and the FMR1-CGG19 and FMR1-1AGGb substrates (panel B). The stiffness of the repeat tract abruptly changes at the interruption, which is indicated by *.

(3) Packer, M. J., Dauncey, M. P., and Hunter, C. A. (2000) Sequence-dependent DNA structure: tetranucleotide conformational maps, *J Mol Biol 295*, 85–103.

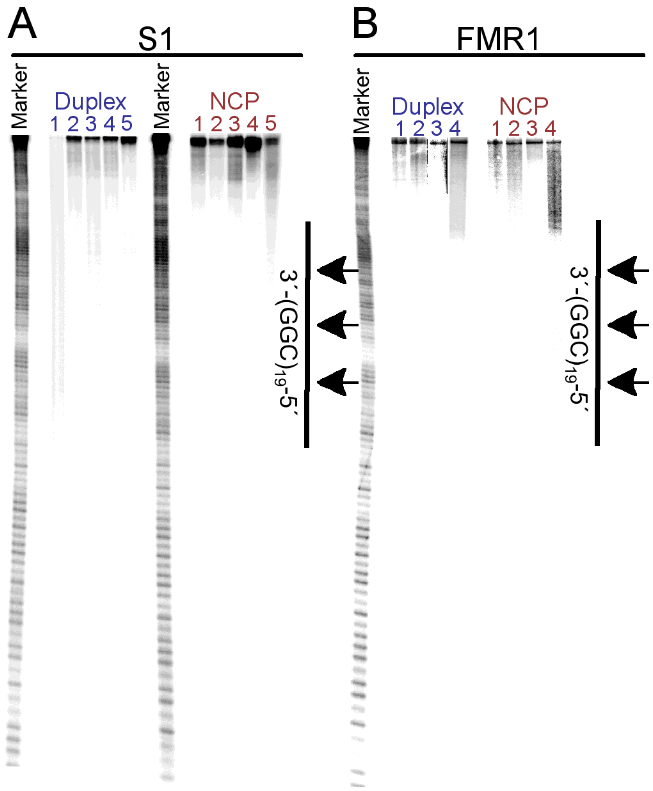


Supporting Information Figure S3: S1 nuclease digestion reveals no single stranded regions in the S1 or FMR1 DNA substrates. Radiolabled DNA substrates were incubated with S1 nuclease, a probe for single-stranded DNA. Reactions were performed with both free duplex (labeled in blue) and nucleosome (NCP, labeled in red) substrates. Reaction with the S1 substrates is shown in panel A, while reaction with the FMR1 substrates are shown in panel B. In panel A, lanes 1 contain the S1 control, lanes 2 contain S1-CGG19, lanes 3 contain S1-1AGGa, lanes 4 contain S1-1AGGb, and lanes 5 contain S1-2AGG. In panel B, lanes 1 contain FMR1-CGG19, lanes 2 contain FMR1-1AGGa, lanes 3 contain FMR1-1AGGb, and lanes 4 contain FMR1-2AGG. In both panels, the marker lane consists of the Maxam-Gilbert A/G sequencing reaction performed on S1a. The location of the repeat tract and interruptions (arrows) are indicated on the right-hand side of the gel.

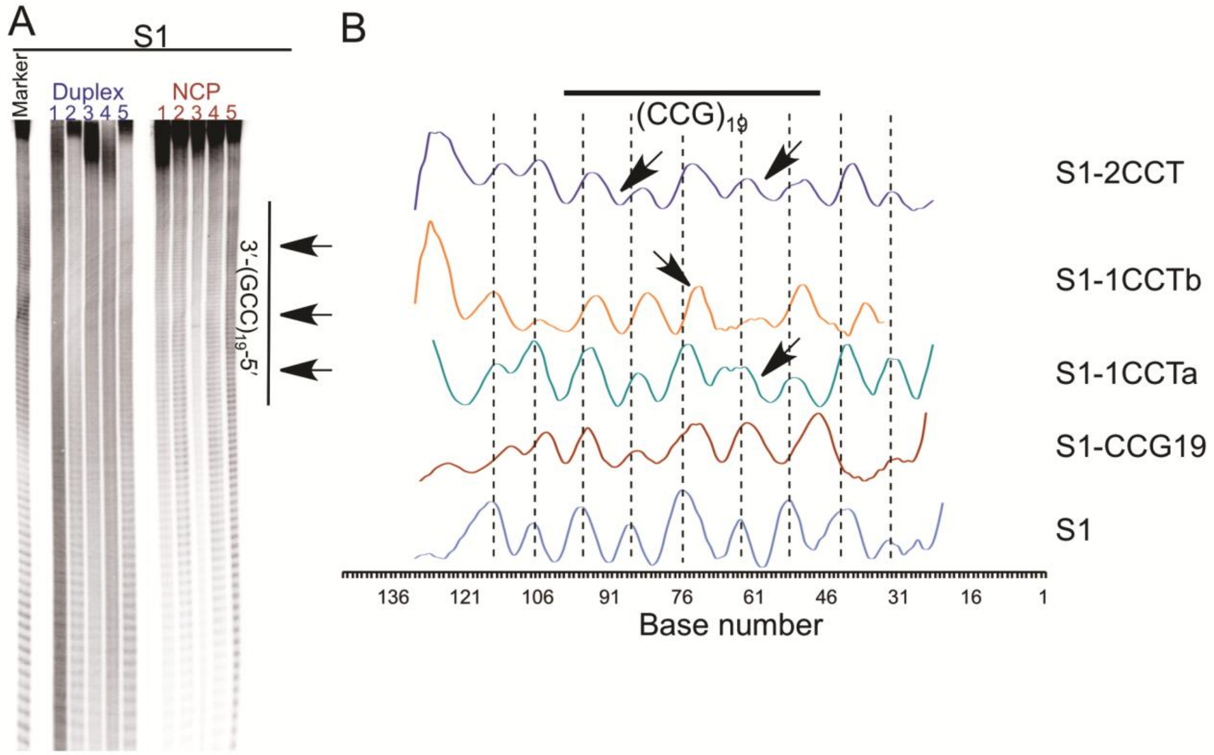


Supporting Information Figure S4: Hydroxyl radical footprinting reveals the periodicity of the DNA around the histone core. Radiolabeled samples, both free duplex (labeled in blue) and nucleosomes (NCP, labeled in red) substrates, were exposed to hydroxyl radicals, revealing a characteristic pattern of oscillating high and low reactivity as the DNA wraps around the histone core. Reactivity toward hydroxyl radical for the CCG-containing strands of the S1 substrates are shown in panel A. Reactions were performed on both free duplex (labeled in blue) and nucleosome (NCP, labeled in red) substrates. In panel A, lanes 1 contain the S1 control, lanes 2 contain S1-CCG19, lanes 3 contain S1-1CCTa, lanes 4 contain S1-1CCTb, and lanes 5 contain S1-2CCT. The marker lane consists of the Maxam-Gilbert A/G sequencing reaction performed on S1b. The location of the repeat tract and interruptions are indicated on the right-hand side of the gel. Panel B contains histograms generated from the gel presented in A. The dashed lines indicate the maxima of the S1 substrate and the arrows indicate the position of the AGG interruptions.

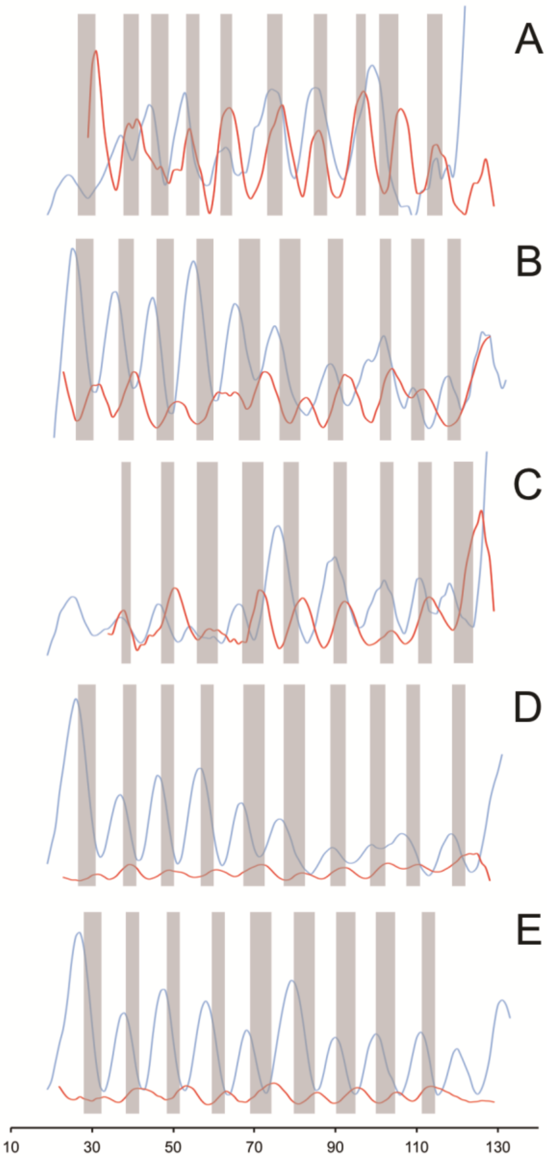


Supporting Information Figure S5: Hydroxyl radical cleavage curves reveal the rotational setting of the DNA around the histone core. The gray bars indicate areas where the minor groove is solvent exposed. Only the S1 series of substrates, S1-CGG19 (A), S1-1AGGa (B), S1-1AGGb (C), S1-2AGG (D), and the S1 control (E) are displayed, as the FMR1 substrates occupy many rotational positions. The S1a or CGG-containing strand is shown in blue (data from Figure 4B) and the S1b or CCG-containing strand is shown in red (data from Supplemental Figure S5B, shown here in the 3′ to 5′ direction).

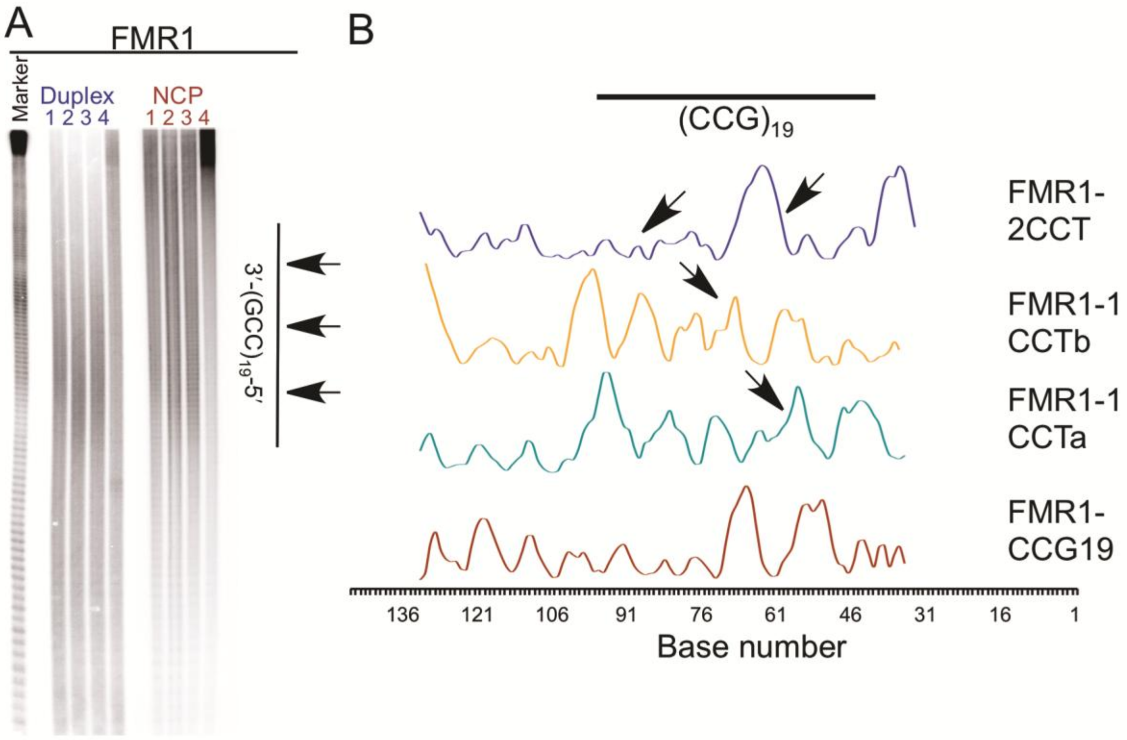


Supporting Information Figure S6: Hydroxyl radical footprinting reveals the periodicity of the DNA around the histone core. Radiolabeled samples, both free duplex (labeled in blue) and nucleosomes (NCP, labeled in red) substrates, were exposed to hydroxyl radicals, revealing a characteristic pattern of oscillating high and low reactivity as the DNA wraps around the histone core. Reactivity toward hydroxyl radical for the CCG-containing strands of the FMR1 substrates are shown in panel A. Reactions were performed on both free duplex (labeled in blue) and nucleosome (NCP, labeled in red) substrates. In panel A, lanes 1 contain FMR1-CCG19, lanes 2 contain FMR1-1CCTa, lanes 3 contain FMR1-1CCTb, and lanes 4 contain FMR1-2CCT. The marker lane consists of the Maxam-Gilbert A/G sequencing reaction performed on S1b. The location of the repeat tract and interruptions are indicated on the right-hand side of the gel. Panel B contains histograms generated from the gel presented in A. The arrows indicate the position of the AGG interruptions.
